# Genome Size Variation in *Dianthus sylvestris* Wulfen sensu lato (Caryophyllaceae)

**DOI:** 10.3390/plants11111481

**Published:** 2022-05-31

**Authors:** Ana Terlević, Sandro Bogdanović, Božo Frajman, Ivana Rešetnik

**Affiliations:** 1 Department of Biology, Faculty of Science, University of Zagreb, Trg Marka Marulića 20/II, 10000 Zagreb, Croatia; ana.terlevic@biol.pmf.hr; 2 Department of Agricultural Botany, Faculty of Agriculture, University of Zagreb Svetošimunska cesta 25, 10000 Zagreb, Croatia; sbogdanovic@agr.hr; 3 Centre of Excellence for Biodiversity and Molecular Plant Breeding, Svetošimunska cesta 25, 10000 Zagreb, Croatia; 4 Department of Botany, Institute of Botany, University of Innsbruck, Sternwartestraße 15, A-6020 Innsbruck, Austria; bozo.frajman@uibk.ac.at

**Keywords:** genome size, Balkan Peninsula, European Alps, tetraploids, glacial refugia

## Abstract

Genome size (GS) is an important characteristic that may be helpful in delimitation of taxa, and multiple studies have shown correlations between intraspecific GS variation and morphological or environmental factors, as well as its geographical segregation. We estimated a relative GS (RGS) of 707 individuals from 162 populations of *Dianthus sylvestris* with a geographic focus on the Balkan Peninsula, but also including several populations from the European Alps. *Dianthus sylvestris* is morphologically variable species thriving in various habitats and six subspecies have been recognized from the Balkan Peninsula. Our RGS data backed-up with chromosome counts revealed that the majority of populations were diploid (2*n* = 30), but ten tetraploid populations have been recorded in *D. sylvestris* subsp. *sylvestris* from Istria (Croatia, Italy). Their monoploid RGS is significantly lower than that of the diploids, indicating genome downsizing. In addition, the tetraploids significantly differ from their diploid counterparts in an array of morphological and environmental characteristics. Within the diploid populations, the RGS is geographically and only partly taxonomically correlated, with the highest RGS inferred in the southern Balkan Peninsula and the Alps. We demonstrate greater RGS variation among the Balkan populations compared to the Alps, which is likely a result of more pronounced evolutionary differentiation within the Balkan Peninsula. In addition, a deep RGS divergence within the Alps likely points to persistence of the alpine populations in different Pleistocene refugia.

## 1. Introduction

Genome size (GS; size of the monoploid chromosome set, [1]) is an important cytogenetic characteristic that may be helpful in delimitation of taxa [2,3,4,5,6,7,8,9,10]. Numerous studies using GS data, often in combination with chromosome counts, have explored diversification of polyploid species complexes [11,12,13,14] or genera with high incidence of polyploidy [15,16,17,18]. Flow cytometric GS estimation has become an established method, as it allows rapid estimation of nuclear DNA content of large numbers of individuals in either absolute or relative units [2,19]. It is a faster and more convenient method for ploidy level estimations compared to conventional chromosome counting, and it can be used for detecting rare cytotypes [20] or to provide evidence of GS intraspecific variability [21].

The existence of intraspecific variation in GS has been acknowledged [7,22,23,24] and reported for many species, e.g., *Festuca pallens* Host [23], *Senecio carniolicus* Willd. [25], *Tephroseris longifolia* (Jacq.) Griseb. & Schenk [26], and *Minuartia verna* (L.) Hiern [8], albeit sometimes argued to be of minor evolutionary relevance [24] or suggested to be a result of an experimental artefact [27]. Nevertheless, this variation can be a result of microevolutionary differentiation and can reflect taxonomic heterogeneity [28]. The reasons for GS variation in the absence of polyploidy may be sought in the increased activity of retrotransposons [29] and the accumulation of retrotransposons and other repetitive elements are considered the main factors of GS increase in angiosperms [7].

Multiple studies have shown correlations between intraspecific variation of GS and morphological or environmental factors, as well as geographical distribution [5,26,30,31,32,33,34,35], but exact causes of this variation, and thus the interpretation of GS heterogeneity, remains a challenging task [35,36,37]. For instance, GS is a characteristic that may be related to the variation in plant phenology [38] and water availability [36], and may affect morphological characteristics such as seed size, nuclear and cell volumes, and duration of mitotic and meiotic cycles [39]. Evidence concerning GS variation across environmental gradients may point to the involvement of GS in adaptive evolution [18,34] or speciation and diversification [40]. Thus, GS data can facilitate taxon delimitation at sectional, specific, and intraspecific levels [3,4,6,9,12,15].

*Dianthus* is one of the largest genera of Caryophyllaceae comprising over 300 species distributed throughout Eurasia and northern Africa [41]. This high diversity is a result of rapid radiation and diversification, which was pronounced especially in the Mediterranean Basin [42], where several polymorphic *Dianthus* groups with high intraspecific diversity, and thus unresolved taxonomy exist [43,44,45,46,47,48]. One of them is *Dianthus sylvestris* Wulfen s.l., which is one of the most taxonomically challenging groups of the European flora [49,50]. Its main diversity centers are the Balkan and the Apennine Peninsulas, where large morphological variation has led to description of several taxa growing in various habitats from the Mediterranean coast to the alpine belt [44,45,47,51]. Due to high morphological variability and subtle morphological transitions, there is a dispute concerning the number of taxa. Several authors have tried to develop a sensible intraspecific classification for *D. sylvestris* on the Balkan Peninsula [52,53,54,55,56], leading to recognition of six subspecies [57,58,59]: *D. sylvestris* subsp. *alboroseus* F.K. Mey., *D. sylvestris* subsp. *bertisceus* Rech. f., *D. sylvestris* subsp. *kozjakensis* Micevski, *D. sylvestris* subsp. *nodosus* (Tausch) Hayek, *D. sylvestris* subsp. *sylvestris,* and *D. sylvestris* subsp. *tergestinus* (Rchb.) Hayek. The morphometric study of Terlević et al. (submitted) [60] performed on 97 populations of *D. sylvestris* s.l. across its range on the Balkan Peninsula showed that the states of several morphological traits deemed diagnostic for subspecies, i.e., number and shape of epicalyx scales, calyx length, petal characteristics, and indumentum density, frequently overlap, making the reliable identification of subspecies often difficult and ambiguous. However, the combination of morphological characteristics (i.e., entire or slightly eroded petals and usually one pair of epicalyx scales) and different flowering time clearly distinguishes the thermophilus *D. sylvestris* subsp. *tergestinus* from all other subspecies including the sympatric *D. sylvestris* subsp. *sylvestris* and *D. sylvestris* subsp. *nodosus*.

Within *Dianthus*, the most frequent chromosome number is diploid (2*n* = 2*x* = 30) [61,62,63,64], although polyploid taxa including series with up to seven ploidy levels have been documented (2*n* = 2*x*, 3*x*, 4*x*, 5*x*, 6*x*, 8*x,* and 12*x*) [11,65]. In the Chromosome Counts Database (CCDB) [63], chromosome numbers for 162 *Dianthus* taxa have been registered, of which 89 (55%) are diploid (2*n* = 30), 17 (10%) tetraploid (2*n* = 60), and 11 (7%) hexaploid (2*n* = 90), whereas for 45 taxa (28%) multiple ploidy levels have been recorded. The karyological features of *Dianthus* chromosomes have been rarely reported due to their small size (0.6–2.7 μm long) and large number in polyploids, but most of them are metacentric and of similar size [61,66,67,68].

For *D. sylvestris* two ploidy levels have been reported: diploids (2*n* = 30) throughout the distribution area [61,62,69] and tetraploids (2*n* = 60) from Gorges de Daluis in the Maritime Alps in France [70]. For diploids from Mt. Jahorina in Bosnia and Herzegovina, the GS of 1C = 0.61 pg has been reported by Siljak-Yakovlev et al. [69] and consequently by Pellicer & Leitch [64]. Due to the low number of chromosomally investigated populations, precise information about the incidence of polyploidy within *D. sylvestris* remains unclear and it is unknown how both ploidies, as well as GS variation, correlate to taxonomic entities within the species and if there is a geographic pattern of GS variation that could be of evolutionary significance.

The main aim of this study was thus to investigate GS and ploidy-level variation within *D. sylvestris* s.l., with a geographic focus on the Balkan Peninsula and to a lesser extent the Alps. To this end, we intersect the GS data of 162 populations, calibrated with chromosome counts, with taxonomic entities and explore its geographic variation. More specifically, we (i) ask if there are polyploid populations present in the area and how they are distributed, (ii) explore whether the pattern of relative genome size (RGS) variation correlates to current taxonomic treatment and geography, and (iii) investigate if there is a relationship between RGS and environmental variation. The obtained data, together with other evidence, will help to disentangle the complex relationships within *D. sylvestris* s.l.

## 2. Results

### 2.1. Chromosome Numbers

**Figure 1:**
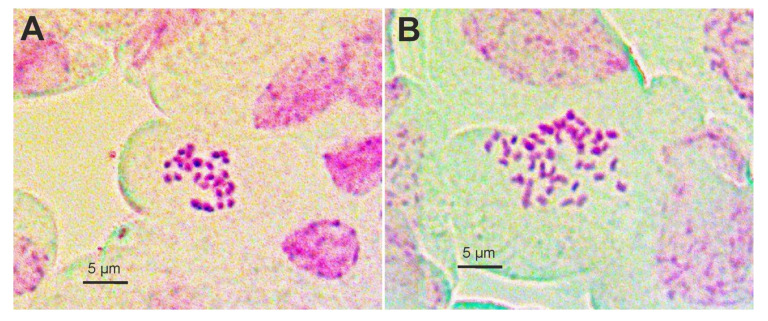
Mitotic chromosomes from *Dianthus sylvestris* root tips. (**A**) A diploid (2*n* = 2*x* = 30) from the population D185 (Punat, Krk island, Croatia) and (**B**) a tetraploid (2*n* = 4*x* = 60) from the population D12 (Plomin, Mt Učka, Croatia).

We estimated the diploid chromosome numbers 2*n* = 2*x* = 30 for two populations from Karlobag (D32) and Krk island (D185) in Croatia and the tetraploid numbers 2*n* = 4*x* = 60 for three populations (D12, D20, and D21) from Istria (Italy and Croatia; Figure 1, Table S1). Chromosomes were small, 1–2 µm long.

### 2.2. Relative Genome Size Estimation and DNA Ploidy Level

**Figure 2:**
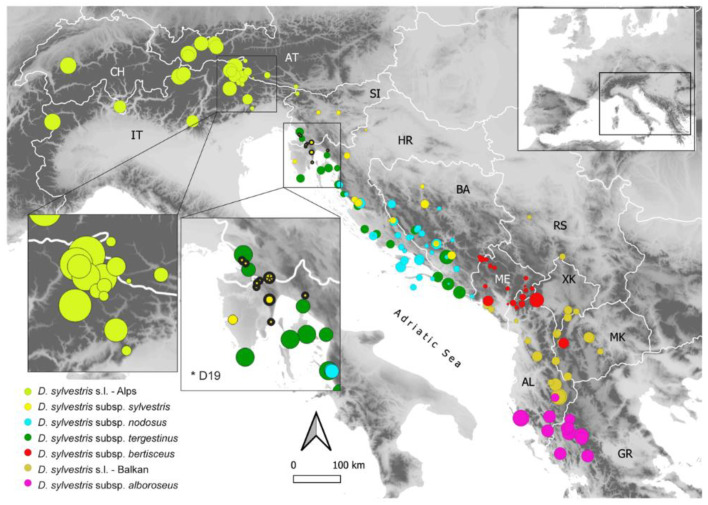
Geographical distribution of the monoploid relative genome size (RGS) variation of 162 diploid and tetraploid (circles with thick black outline) populations of *Dianthus sylvestris* s.l. in the Balkan Peninsula and the Alps. Only populations with at least three measured individuals and a standard deviation of RGS < 0.01 are shown. The size of the dots is proportional to the mean monoploid RGS of the corresponding populations. Asterisk indicates the ploidy-mixed population D19. Color coding indicates different taxa.

**Figure 3:**
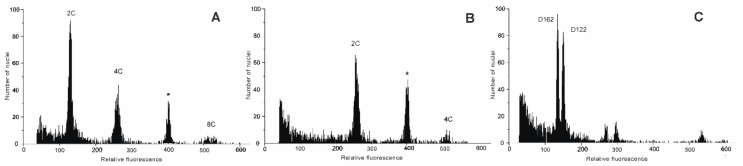
Histograms of fluorescence intensities of diploid (**A**) (population D19) and tetraploid (**B**) (population D17) accessions of *D. sylvestris*, together with the internal reference standard *Bellis perennis* (asterisk), from which relative genome size (RGS) was calculated. The peaks are labelled following Greilhuber et al. [1]. Multiple peaks of the sample correspond to nuclei after one (4C) and two (8C) rounds of endoreplication. (**C**) Histogram showing genuine difference between diploid populations D162 (0.325 ± 0.001) and D122 (0.360 ± 0.004).

RGS was analyzed for 707 individuals from 162 populations of *D. sylvestris* s.l. from the Balkan Peninsula and the Alps (Figure 2, Figure S1). High-resolution histograms of DNA content comprised two large G1 peaks representing nuclei of the sample and the reference (Figure 3), and the ratio of their positions determined the sample’s RGS. In addition to the main peaks, the minor peaks of the sample corresponded to endopolyploid nuclei [71] that are common in Caryophyllaceae [72]. The coefficient of variation (CV) of the sample’s G0/G1 peak of the majority of 707 measurements was between 1.36 to 6 (4.7 on average). In further analyses, we also included 17 populations that exceeded this threshold and had a CV of up to 10, as their peaks were clearly visible and their RGS values fitted well to the remaining data.

**Figure 4:**
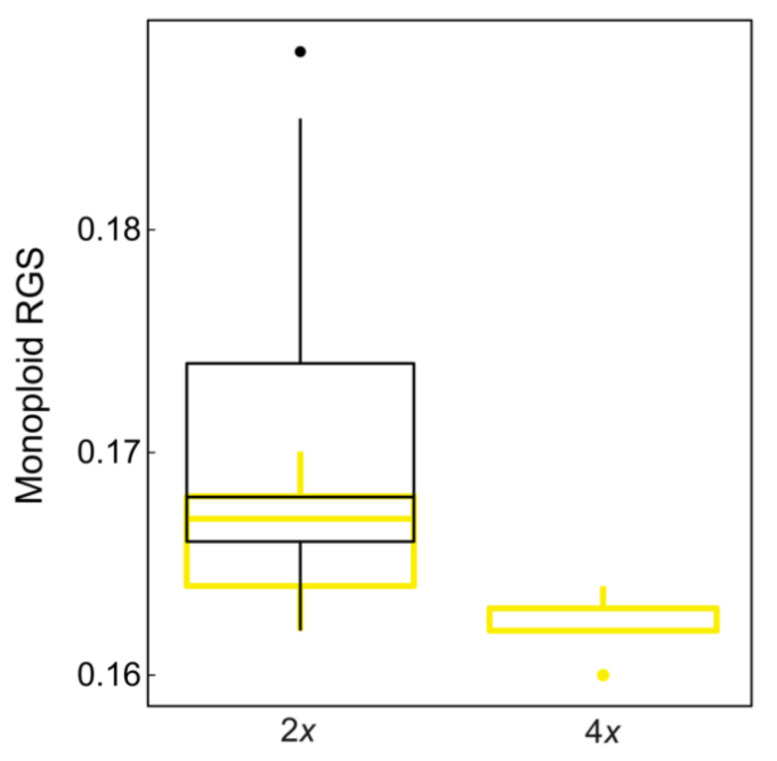
Monoploid relative genome size (RGS) variation in *Dianthus sylvestris* s.l. in the Balkan Peninsula and the Alps. Black colored box indicates the variation of the complete diploid dataset (152 DNA-diploids, 2*x*), whereas yellow color corresponds to 15 DNA-diploids (2*x*), and 10 DNA-tetraploid (4*x*) populations of *D. sylvestris* subsp. sylvestris. Boxes correspond to 25 and 75 percentiles, horizontal lines to medians, whiskers 5 to 95 percentiles, and circles to outliers.

Flow-cytometry screening resulted in two discrete groups of RGS values that corresponded to the estimated diploid and tetraploid chromosome numbers. Diploid populations occurred throughout the sampled area and all subspecies, at elevations from 8 to 2274 m. On the other hand, all tetraploid populations belonged to *D. sylvestris* subsp. *sylvestris*, and were limited to Istria and Kvarner (Croatia and Italy), from 240 to 941 m (Figure 2). A total of 152 populations (94%) were DNA-diploid, with RGS ranging from 0.324 to 0.376 (mean: 0.341 ± 0.011), whereas ten populations (6%) were DNA-tetraploid with RGS ranging from 0.640 to 0.657 (mean: 0.649 ± 0.006; Table 1). A 1.16-fold variation in RGS was thus revealed among diploids with a 1.03-fold variation among tetraploids (Figure 3C, Table 1). Only one population from Istria (Vodice-D19; Figure 2) was ploidy-mixed, with two individuals being DNA-diploid and eight DNA-tetraploid. The monoploid RGS of 15 DNA-diploid populations of *D. sylvestris* subsp. *sylvestris* ranged from 0.162 to 0.188 and was significantly higher compared to ten DNA-tetraploid populations with values between 0.160 and 0.164 (Figure 4, Kruskal–Wallis test = 10.58, *p* < 0.01).

### 2.3. Morphological and Environmental Differences between Diploids and Tetraploids

**Figure 5:**
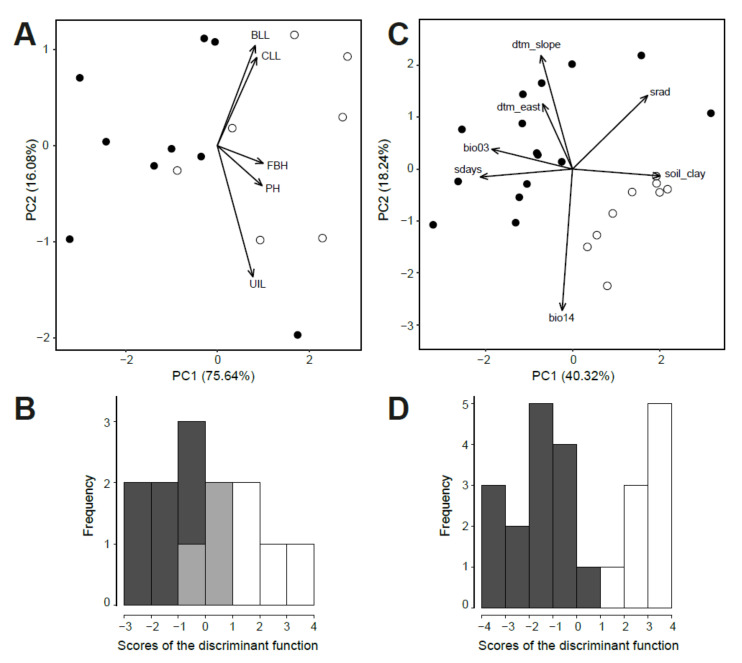
Morphological and environmental differentiation between diploid (black) and tetraploid (white) populations of *Dianthus sylvestris* subsp. *sylvestris*, with their overlap in B shown in grey. (**A**) Principal component analysis (PCA) and (**B**) histogram of discriminant analysis (DA) based on five morphological characteristics. (**C**) PCA and (**D**) histogram of DA based on seven environmental variables. Characteristic abbreviations in (**A**,**C**) are explained in Tables S2 and S3.

Comparison of morphological characteristics from the study of Terlević et al. (submitted) [60] between the nine diploid and seven tetraploid populations of *D. sylvestris* subsp. *sylvestris* showed a statistically significant difference in five vegetative characteristics (Table S2). The first two PCA axes explained 75.64% and 18.06% of the total morphological variation (Figure 5A) and the characteristics contributing most to the separation along the first axis were plant height (PH) and height of the first branching (FBH; component scores 0.49 and 0.50). The same variables had the highest scores in the DA (0.80 and 0.93), even though there was overlap between the scores of the discriminant functions (Figure 5B). The tetraploid plants were thus higher and had their lowermost lateral shoots higher. In addition, they had longer cauline and basal leaves (CLL, BLL), as well as longer internodes (UIL).

Although the correlation test failed to show any association between RGS of 152 diploid populations and environmental variables, the Kruskal–Wallis test showed significant difference in 14 environmental variables between 15 diploid and ten tetraploid populations of *D. sylvestris* subsp. *sylvestris* (Table S3 and Figure S2). The environmental variables contributing most to the separation of diploid and tetraploid populations were those describing temperature and precipitation preferences, as well as the terrain geomorphology. The first two PCA axes explained 40.32% and 18.24% of the total environmental variation (Figure 5C) and the variables contributing most to the separation along the first axis were soil clay content and number of snow days in a year (component scores 0.50 and −0.53). Number of snow days in a year, slope and eastness had the highest scores in the DA (−0.59 and −0.64), without overlap between diploids and tetraploids (Figure 5D).

### 2.4. RGS Variation across Intraspecific Entities

**Figure 6:**
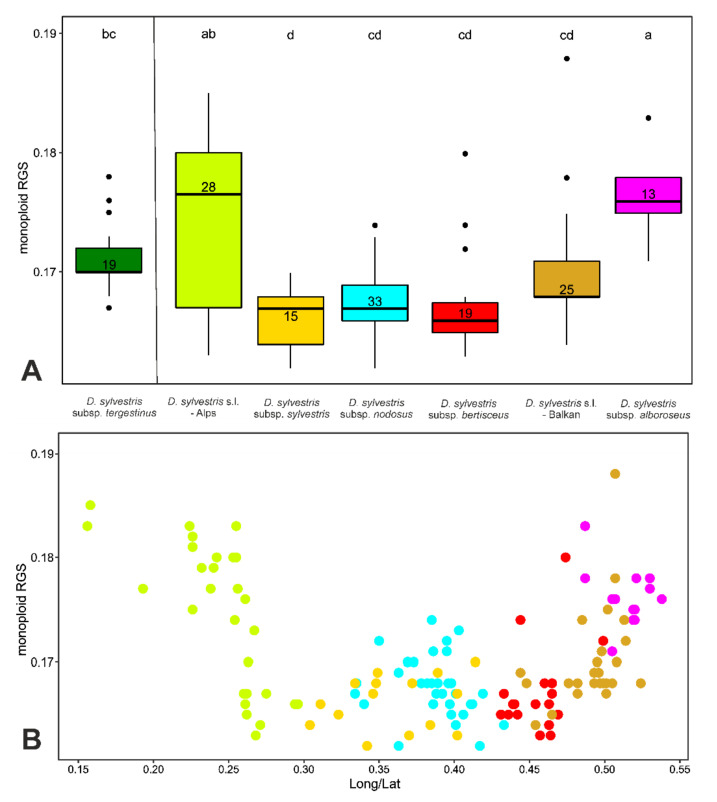
Monoploid RGS variation based on population means of 152 DNA-diploid populations of *Dianthus sylvestris* s.l. Color coding for taxa corresponds to Figure 2. (**A**) Boxes in the boxplot and (**B**) points in the scatterplot are arranged in geographical order from the north-west to the south-east. Being the most clearly distinguished subspecies, D. sylvestris subsp. tergestinus is singled out on the left of plot (**A**) and excluded from the plot (**B**). Boxes correspond to 25 and 75 percentiles, horizontal lines to medians, whiskers 5 to 95 percentiles, and circles to outliers. Means not significantly different at *p* < 0.01, according to the Tukey post-hoc test, are indicated by the same letter. Numbers adjacent to the median lines represent the sample size.

Differences in RGS among the five subspecies and two geographic groups of populations of *D. sylvestris* s.l. were significant (Kruskal–Wallis = 54.1, *p* < 0.01, Figure 6A). The Tukey post-hoc test showed that populations of *D. sylvestris* subsp. *alboroseus* and those of *D. sylvestris* s.l. from the Alps had significantly larger monoploid RGS values than other groups (*p* < 0.01, Figure 6A). Additionally, the RGS of *D. sylvestris* subsp. *tergestinus* was significantly higher than the RGS of *D. sylvestris* subsp. *sylvestris,* whereas there was no significant difference between *D. sylvestris* s.l. from the Balkans, *D. sylvestris* subsp. *bertisceus, D. sylvestris* subsp. *nodosus,* and *D. sylvestris* subsp. *sylvestris.*

## 3. Discussion

### 3.1. Tetraploidization within D. sylvestris Populations in the Northern Balkan Peninsula

The extensive RGS measurements combined with confirmatory chromosome number estimations revealed the prevalence of diploid populations of *D. sylvestris* throughout the investigated area, as well as the occurrence of tetraploid populations in the northwesternmost Balkan Peninsula (Istria and Kvarner in Croatia and Italy; Figure 2, Table S1). Therefore, this is the first report of tetraploid populations within *D. sylvestris* s.l. in the Balkan Peninsula, which likely originated separately from the tetraploid populations reported from France [70], given the geographic distance between them. The confirmation of independent origin of these two groups of tetraploid populations requires additional evidence based on genetic data or detailed examination of RGS values of French populations as their different values might indicate a separate origin. However, multiple polyploidization events within single species are common and have been reported, for example in *Astragalus onobrychis* [73], *Cerastium decalvans* [74], and *Euphorbia montenegrina* [10]. In addition, within *Dianthus*, multiple and independent origin of polyploids within the *Dianthus broteri* complex [75,76] and several heteroploid species of *Dianthus* section *Plumaria* [11], that even occur sympatrically, have been reported. Diploidization, a process following polyploidization, is commonly accompanied by elimination of parts of the genome [77,78], termed genome downsizing [79]. Reduction of monoploid GS has been observed in many different plant groups (e.g., [8,15,79,80]) and our data suggest that it is occurring also in tetraploid *D. sylvestris*, as its monoploid RGS was significantly smaller compared to the diploids (Kruskal–Wallis test = 10.58, *p* < 0.01).

Given the morphological similarity of tetraploid populations to their diploid counterparts occurring in the same area, both being identified as *D. sylvestris* subsp. *sylvestris*, we suggest an autopolyploid origin of tetraploids, as it was also suggested for polyploids within *Dianthus* sect. *Plumaria* [11]. A tetraploid origin from the diploid *D. sylvestris* subsp. *sylvestris,* rather from *D. sylvestris* subsp. *tergestinus* that also occurs in the same area (Figure 2), is further supported by more similar monoploid RGS of the tetraploids with the former taxon. Alternatively, an allopolyploid origin involving putative diploid parents of northern i.e., alpine and eastern i.e., Balkan provenance is possible. Despite a clear identification of tetraploid populations as *D. sylvestris* subsp. *sylvestris*, we demonstrated that tetraploid individuals differ from diploids in stem and leaf size characteristics. This is in line with other studies, where it has been shown that polyploidization can substantially affect morphological variation and, although the relationship between ploidy and body/organ size is complex, polyploid plants are often larger than their diploid parents [81,82].

In addition to morphological divergence, ecological differentiation among different ploidies within the same species has also been evidenced [76,83,84]. Given that polyploids may be more competitive compared to diploids [85], they are expected to have higher dispersal potential and may thrive in different ecological niches. It has been suggested that diploids tend to be restricted to refugia, whereas polyploids show better ability to re-colonize deglaciated regions [86]. However, contradicting cytogeographic patterns were also observed [8,87], and in *D. sylvestris* the tetraploids were also found to be geographically and environmentally restricted compared to the diploids that thrive in a broad range of environments. Diploid and tetraploid populations of *D. sylvestris* subsp. *sylvestris* were environmentally segregated by different temperature and precipitation preferences, as well as different geomorphology of the terrain. Tetraploids were collected in warmer habitats with less fluctuation in temperature, and more precipitation during the driest month. Furthermore, they thrive on significantly less steep, westerly exposed slopes with higher clay content in the soil and in habitats with a lower number of frost and snow days per year and with higher surface solar radiation. Nevertheless, one mixed-ploidy population from north-west Croatia (Vodice-D19) provides evidence that tetraploids and their diploid progenitors can occupy similar environments [88] and further studies are needed to reveal whether the observed ecological divergence is adaptive or simply a result of much wider distribution of diploids inhabiting a broader array of environments. Interestingly, the area of Istria, where tetraploids occur, is known as an important Pleistocene refugium for plants [89,90]. It is likely that Pleistocene climatic oscillation also triggered polyploidization in *D. sylvestris*, given the Pleistocene radiation in Eurasian *Dianthus* [42].

### 3.2. Geographic and Intraspecific Variation of RGS within Diploid D. sylvestris s.l.

Despite the fact that RGS estimations were performed with DAPI, which is an AT-content dependent, and cannot be, with 100% reliability, translated to absolute values and thus used in comparative studies [91], such a conversion based on the GS of our standard *Bellis perennis* (2C = 3.38 pg, [92]) revealed a variation of 1C in *D. sylvestris* s.l. ranging between 0.55 and 0.64 pg (mean: 0.58 pg, N = 152 populations). These values therefore correspond well to the estimated absolute GS of 1C = 0.61 pg [69]. *Dianthus sylvestris* is thus a small-genome species and it has been suggested that plants with small genomes display more pronounced morphological variation and thrive in wide environmental gradients, compared to large-genome taxa [37]. *Dianthus sylvestris* exhibits a high morphological variability and grows from the coastal Mediterranean environment to the alpine belt both in the Alps as well as the Balkan Peninsula [60] and hence supports the available evidence, but further studies across flowering plants are needed to bring more evidence for this hypothesis.

Our comprehensive sampling revealed an indicative spatial pattern of RGS variation within diploid populations of *D. sylvestris* (Figure 2 and Figure 6B). Although the general pattern is complex and often populations with clearly different RGS occur in vicinity, larger RGS values predominate in the southern Balkan Peninsula and the central part of the Alps, i.e., at the south-eastern and northern margin of the species distribution. A clear geographic pattern in RGS variation with smaller monoploid RGS in the distribution center and its increase towards the distribution margins were also observed at the genus level in *Knautia* [15] and *Sesleria* [16]. Causes for such patterns remain unclear, but it has been suggested that larger GS can limit adaptive and competitive abilities of populations at the distribution margins and might thus represent a factor limiting further range expansion [37], however further studies are needed to rigorously test this hypothesis.

The southernmost populations with large RGS correspond to *D. sylvestris* subsp. *alboroseus.* Therefore, this subspecies also exhibited highest RGS among the Balkan taxa (Figure 6B). Towards the north-west, the RGS decreases in populations morphologically intermediate between *D. sylvestris* subsp. *alboroseus* and *D. sylvestris* subsp. *bertisceus* and thus not clearly classified to any subspecies and reaches the smallest values in *D. sylvestris* subsp. *bertisceus*. Further to the north-west, a slight, although statistically non-significant, increase in geographically adjacent *D. sylvestris* subsp. *nodosus* and *D. sylvestris* subsp. *sylvestris* can be observed. The latter two subspecies can hardly be distinguished morphologically, they have similar environmental niches [60] and their highly similar RGS renders their recognition as two subspecies questionable. Interestingly, these two subspecies with similar morphology and RGS appear to form a unique phylogenomic cluster based on preliminary analyses of the RADseq data (Temunović et al., unpublished), separated from more southern populations, which are genetically more diverse. More pronounced genetic differentiation of southern compared to northern populations has been observed in several plant groups (e.g., [10,74,93,94,95]) and is, in *D. sylvestris*, also reflected in more pronounced RGS variation in this geographic region. Along the same line, ecologically and morphologically distinct *D. sylvestris* subsp. *tergestinus* [60] growing along the Adriatic coast, had higher RGS compared to geographically partly sympatric *D. sylvestris* subsp. *nodosus* and *D. sylvestris* subsp. *sylvestris*. Preliminary analyses of the genomic RADseq data (Temunović et al., unpublished) suggests that *D. sylvestris* subsp. *tergestinus* forms an evolutionary lineage distinct from all other Balkan populations of *D. sylvestris*. Its divergent RGS, which is significantly different from *D. sylvestris* subsp. *sylvestris*, is thus likely a result of divergent evolutionary histories.

Also, in the Alps, there is a pronounced variation in RGS within *D. sylvestris*, with a clear trend in its geographical distribution (Figure 2). Whereas the majority of the analyzed samples scattered across the western parts of the Eastern Alps, and a few populations from the Western Alps, exhibit larger RGS ranging between 0.346 and 0.369, the easternmost alpine populations (most of them from the eastern part of the Southern Alps) have smaller RGS ranging between 0.326 and 0.339 that correspond to the RGS of the populations from the north-western Balkan Peninsula. Therefore, the most prominent RGS variation among all groups analyzed was within the alpine group (Figure 6A). This relatively abrupt change in RGS within the Alps could be a result of divergence due to persistence of the species in two (or more) separate glacial refugia. It is likely that the eastern populations from the Southern Alps shared their refugium with the northern Balkan populations of *D. sylvestris* subsp. *sylvestris* in the north-western Balkan Peninsula, whereas the other alpine populations with clearly higher RGS survived the glacial cycles in more western refugia; several isolated refugia along the southern margin of the Alps have also been suggested for other alpine plants by Schönswetter et al. [96]. The observed RGS divergence in *D. sylvestris* within the Alps corresponds to a genetic discontinuity (Luqman et al., unpublished; Temunović et al., unpublished), but the exact border between the two lineages and their relation to the GS remains to be determined. In South Tyrol and adjacent Veneto (Italy) as well as East Tyrol (Austria), populations with relatively high RGS grow in close vicinity with the populations with lower RGS, suggesting that there might be a hybrid zone between the alpine and the Balkan lineage.

Multiple studies have suggested that environmental conditions may place constraints on the evolution of GS [34,37], hence genome size can either be directly associated with temperature and precipitation [97] or indirectly associated through elevation [34] or latitude [17,37,98]. However, the lack of association of RGS with environmental variables in our study on one hand, and a clear geographic trend in the RGS variation in *D. sylvestris* on the other hand, suggest that it was likely the phylogeographic rather than the environmental divergence that shaped the RGS variation in our study species.

## 4. Materials and Methods

### 4.1. Plant Material

We sampled 134 populations of *D. sylvestris* s.l. throughout the Balkan Peninsula and 28 populations from the European Alps between 2018 and 2021. At each locality, basal leaves from 3 to 12 individual plants were desiccated in silica gel for RGS measurements, and an herbarium specimen was collected. For chromosome number estimations, we collected mature seeds from several localities in July and August of 2020 and 2021. The seeds were air-dried and dry-stored in darkness at room temperature. Vouchers are deposited in the herbarium ZA (Table S1). We identified the plants and assigned them to subspecies based on identification keys in national and regional floras [44,99,100,101,102,103,104,105], and treated the populations that we could not assign to any of the known subspecies based on their morphology as *D. sylvestris* s.l.

### 4.2. Chromosome Counts

We determined chromosome numbers for five populations (Table S1). The seeds were germinated at the surface of a peat medium in plastic pots with regular watering at room temperature. Root tips were harvested at about noon and pre-treated with 0.002 M 8-hydroxyquinoline for 4 h in darkness at 4 °C. Subsequently, material was fixed in 3:1 ethanol–glacial acetic acid for 12–24 h at 4 °C. The fixed root tips were hydrolyzed in 5 M HCl at room temperature for 45 min, and then washed in distilled water. The root tips were stained in Schiff’s reagent for 2 h. Finally, we squashed the stained root tips on a slide glass in a drop of 45% (*v*/*v*) acetic acid. Photomicrographs of chromosomes at mitotic metaphase were taken with Zeiss Lab. A1 AXIO microscope (Carl Zeiss Microscopy, Jena, Germany) equipped with ToupCam 5.1 MP digital camera. Snapshots were exported and studied using ImageJ software.

### 4.3. Flow Cytometry

Silica-gel-dried leaves were analyzed using flow cytometry (FCM) of 4′,6-diamidino-2-phenylindole (DAPI; final concentration 0.036 M) stained nuclei [106] to estimate RGS and DNA ploidy levels of sampled populations. We used *Bellis perennis* as the primary internal standard [92]. Desiccated green leaf tissue (c. 0.5 cm^2^) of one to two plant individuals from the same population was chopped together using a sharp razor blade in a plastic Petri dish, with an appropriate amount of fresh reference standard and processed as described in Suda et al. [20]. The relative fluorescence intensity of 3000 nuclei was recorded using a Partec CyFlow Space flow cytometer (Sysmex Partec, Münster, Germany). We used Partec FloMax software to evaluate histograms and to calculate coefficients of variation (CV) of the standard and sample peaks. We calculated RGS as the ratio between the mean relative fluorescence of sample and standard. Samples with CV of the G1 peak >10% were re-analyzed until sufficient quality was achieved [21]. The number of measured individuals per population yielding high quality FCM histograms is given in Table S1.

### 4.4. Statistical Analyses

We calculated mean RGS value and standard deviation for each population from individual measurements of at least three individuals and we inferred the DNA ploidy levels [107] for all analyzed populations. We performed the Kruskal–Wallis test and Tukey post-hoc tests on population means, to evaluate statistically significant differences. The difference in monoploid RGS between diploids and tetraploids was tested for significance on a subset of 25 populations of *D. sylvestris* subsp. *sylvestris*, whereas the difference in RGS among the subspecies was tested for all diploid populations (N = 152). Due to the very small sample size (only one population known from the locus classicus), *D. sylvestris* subsp. *kozjakensis* was not included in these tests. All statistical analyses were performed using R-4.0.2 [108].

We performed principal component analysis (PCA) and discriminant analysis (DA) to explore the variability and the relative importance of characteristics/variables discriminating between the diploid and the tetraploid populations of *D. sylvestris* subsp. *sylvestris*. Both PCA and DA were performed for the morphological and the environmental dataset. The morphometric PCA and DA were performed using five morphological characteristics showing significant difference between the two ploidy levels (Table S2), whereas the environmental PCA and DA were based on seven environmental variables showing significant difference between the ploidy levels (Table S3) and without collinearity issues.

We used morphological data of the populations from the Balkan Peninsula from Terlević et al. ([60], Table S1) and tested the differences between nine diploid and seven tetraploid populations of *D. sylvestris* subsp. *sylvestris* (N = 16). We downloaded environmental data from three databases: climate data from the Chelsa database [109,110], soil properties from the SoilGrids [111], and topographic variables from the EarthEnv database [112]. The environmental space of each studied population was depicted by extracting environmental data from the points defined by longitude (N) and latitude (E; Table S1), and the differences were tested between 15 diploid and 10 tetraploid populations of *D. sylvestris* subsp. *sylvestris* (N = 25). The association between DNA-ploidy levels and environmental variables was also visualized with the package ‘ggplot2′. Environmental variables were standardized to meet the assumption of homogeneity of variance and linearity. The correlation between environmental and RGS variability of 152 diploid populations in the studied area was tested employing Pearson correlation coefficients.

## 5. Conclusions

By analyzing the RGS variation of *Dianthus sylvestris* in the Balkan Peninsula and to a lesser extent in the Alps, our results reveal complex patterns of RGS in widespread diploid populations and spatially restricted tetraploid populations in the north-western Balkan Peninsula. The populations in the central and western parts of the Alps, as well as those at the southern distribution limit in the Balkan Peninsula, exhibit higher RGS, likely corresponding to discrete evolutionary lineages. In addition, two areas of more pronounced RGS variation at small geographic distances are revealed. One is located in the eastern Alps and the other in southern Dinaric Mountains. We suggest that the observed RGS differences are a result of evolutionary divergence due to persistence in separate glacial refugia. However, only upcoming integration of phylogenomic data will show the correlation of observed morphological (taxonomic) and GS variation with evolutionary differentiation.

## Figures and Tables

**Table 1:** Descriptive statistics of relative genome size (RGS) variation in diploid (2*x*) and tetraploid (4*x*) populations of *Dianthus sylvestris* s.l., for which at least three individuals were analyzed. SD, standard deviation; mRGS, monoploid RGS.

Ploidy	No. Measurements	Populations	Individuals	Min. No. Individuals	Max. No. Individuals	Mean No. Individuals	Mean RGS	SD RGS	Min RGS	Max RGS	Mean mRGS	SD mRGS
2*x*	536	152	657	3	12	4.3	0.341	0.011	0.324	0.376	0.17	0.005
4*x*	45	10	50	3	8	5	0.649	0.006	0.64	0.657	0.162	0.001

## Data Availability

Data are contained within the article or the Supplementary Materials.

## Supplementary Materials

The following supporting information can be downloaded at: https://www.mdpi.com/article/10.3390/plants11111481/s1. **Table S1.** Relative genome size (RGS) and ploidy level (2*x*, DNA-diploids; 4*x*, DNA-tetraploids) of 162 populations of *Dianthus sylvestris* from the Balkan Peninsula and the Alps, including their provenance and voucher data. For each population, DNA ploidy estimates, mean RGS of a holoploid genome and standard deviation are given. Number of individuals analyzed for RGS in each population (N) and the range of CV values are also provided. Ploidy: 2*x* and 4*x*. Asterisk (*) indicates the populations for which confirmatory chromosome counts were performed. Plus (+) in the column Morphometrics indicates the morphometrically analyzed populations in the previous study by Terlević et al. (submitted) [60]. **Figure S1.** Relative genome size (RGS) in diploid populations of *Dianthus sylvestris* sorted by increasing RGS values. Population means (dots) with corresponding standard deviation (vertical lines) are presented. Population names correspond to Table S1. Colors correspond to subspecies, as in Figure 2. **Table S2.** Results of Kruskal–Wallis test between diploid (2*x*, N = 9) and tetraploid (4*x*, N = 7) populations of *Dianthus sylvestris* subsp. *sylvestris*. Morphological characteristics with significant differences between ploidy levels at *p* < 0.05 are shown in bold. **Table S3.** Results of Kruskal–Wallis test between diploid (2*x*, N = 15) and tetraploid (4*x*, N = 10) populations of *Dianthus sylvestris* subsp. *sylvestris*. Environmental variables with significant differences between ploidy levels at *p* < 0.05 are shown in bold. **Figure S2.** Boxplots showing environmental differences along the 14 environmental variables between diploid (2*x*) and tetraploid (4*x*) populations of *Dianthus sylvestris* subsp. *sylvestris*. Only variables with significant differences (*p* < 0.05), as revealed by Kruskal–Wallis test (Table S3), are shown.
