# Supplementary material for: Genome Size Variation in Dianthus sylvestris Wulfen sensu lato (Caryophyllaceae)
Source: Plants (Basel). 2022 May 31;11(11):1481. doi: 10.3390/plants11111481 (PMC9183063; doi:10.3390/plants11111481)
Supplement: Supplementary file 1 [file plants-11-01481-s001.zip › plants-1700177-supplementary.pdf]

Table S1. Relative genome size (RGS) and ploidy level (2*x*, DNA-diploids; 4*x*, DNA-tetraploids) of 162 populations of *Dianthus sylvestris* from the Balkan Peninsula and the Alps, including their provenance and voucher data. For each population, DNA ploidy estimates, mean RGS of a holoploid genome and standard deviation are given. Number of individuals analyzed for RGS in each population (N) and the range of CV values are also provided. Ploidy: 2*x* and 4*x*. Asterisk (*) indicate the populations for which confirmatory chromosome counts were performed. Plus (+) for Morphometrics indicate the morphometrically analyzed populations in the previous study by Terlević et al. (submitted) [60].

ID	Population name	Taxon	Country	Altitude (m a.s.l.)	Long (N)	Lat (E)	Collector	Collection date	Voucher	Mean RGS	SD RGS	N	Range of CV	Ploidy	Morpho-metrics
D131	Pindus Theodoriana	*D. sylvestris* subsp. *alboroseus*	Greece	1023	39.42647	21.21591	S. Bogdanović, I. Ljubičić, I. Rešetnik, A. Terlević	21/06/2019	ZA-54934	0.352	0.003	5	1.99–2.82	2x	
D132	Mt Gorilla	*D. sylvestris* subsp. *alboroseus*	Greece	664	39.49754	20.54052	S. Bogdanović, I. Ljubičić, I. Rešetnik, A. Terlević	21/06/2019	ZA-54935	0.351	0.002	6	2.14–2.46	2x	+
D133	Lygeres Siolades	*D. sylvestris* subsp. *alboroseus*	Greece	744	39.76638	21.09354	S. Bogdanović, I. Ljubičić, I. Rešetnik, A. Terlević	22/06/2019	ZA-54936	0.353	0.003	5	2.15–4.74	2x	+
D134	Lygeres Aoos	*D. sylvestris* subsp. *alboroseus*	Greece	1398	39.82082	21.08762	S. Bogdanović, I. Ljubičić, I. Rešetnik, A. Terlević	22/06/2019	ZA-54937	0.357	0.003	6	2.84–4.43	2x	
D135	Kapesovo	*D. sylvestris* subsp. *alboroseus*	Greece	1135	39.88615	20.78638	S. Bogdanović, I. Ljubičić, I. Rešetnik, A. Terlević	22/06/2019	ZA-54938	0.356	0.002	5	2.84–4.09	2x	+
D137	Pindus Pyrgos	*D. sylvestris* subsp. *alboroseus*	Greece	631	40.14424	20.82399	S. Bogdanović, I. Ljubičić, I. Rešetnik, A. Terlević	22/06/2019	ZA-54940	0.348	0.006	4	2.93–3.84	2x	+
D138	Pindus Mikropapingo	*D. sylvestris* subsp. *alboroseus*	Greece	1296	39.97295	20.74076	S. Bogdanović, I. Ljubičić, I. Rešetnik, A. Terlević	23/06/2019	ZA-54941	0.350	0.003	6	2.02–2.57	2x	+
D139	Pindus Drakolimni	*D. sylvestris* subsp. *alboroseus*	Greece	2093	39.99494	20.78599	S. Bogdanović, I. Ljubičić, I. Rešetnik, A. Terlević	23/06/2019	ZA-54942	0.349	0.005	5	2.77–4.72	2x	
D140	Mt Dhembel	*D. sylvestris* subsp. *alboroseus*	Albania	1523	40.21507	20.31646	S. Bogdanović, I. Ljubičić, I. Rešetnik, A. Terlević	24/06/2019	ZA-54943	0.351	0.000	6	1.98–2.31	2x	+
D141	Gjirokaster	*D. sylvestris* subsp. *alboroseus*	Albania	400	39.94504	20.25109	S. Bogdanović, I. Ljubičić, I. Rešetnik, A. Terlević	25/06/2019	ZA-54944	0.353	0.002	6	2.54–2.59	2x	+
D142-1	Llogara village	*D. sylvestris* subsp. *alboroseus*	Albania	950	40.21436	19.57959	S. Bogdanović, I. Ljubičić, I. Rešetnik, A. Terlević	25/06/2019	ZA-54946	0.355	0.003	4	3.01–4.41	2x	
D142-2	Llogara park	*D. sylvestris* subsp. *alboroseus*	Albania	1600	40.20721	19.59952	S. Bogdanović, I. Ljubičić, I. Rešetnik, A. Terlević	25/06/2019	ZA-54945	0.365	0.010	4	4.43–5.55	2x	
D78	Mt Ostrovice	*D. sylvestris* subsp. *alboroseus*	Albania	1386	40.5741	20.47551	D. Lakušić, N. Kuzmanović, I. Janković, M. Zbiljić	11/07/2018	ZA-48633	0.342	0.004	3	2.46–4.70	2x	
D150	Rrapsh	*D. sylvestris* subsp. *bertisceus*	Albania	773	42.41509	19.50371	S. Bogdanović, I. Ljubičić, I. Rešetnik, A. Terlević	27/06/2019	ZA-54956	0.336	0.005	6	2.19–2.80	2x	+
D151	Thethi Shtegut	*D. sylvestris* subsp. *bertisceus*	Albania	1735	42.38844	19.73021	S. Bogdanović, I. Ljubičić, I. Rešetnik, A. Terlević	28/06/2019	ZA-54957	0.336	0.002	6	3.63–5.02	2x	+
D153	Rruga Thethe	*D. sylvestris* subsp. *bertisceus*	Albania	665	42.32486	19.59345	S. Bogdanović, I. Ljubičić, I. Rešetnik, A. Terlević	28/06/2019	ZA-54959	0.332	0.004	5	3.25–4.62	2x	+
D154	Cijevna	*D. sylvestris* subsp. *bertisceus*	Montenegro	139	42.39835	19.36711	S. Bogdanović, I. Ljubičić, I. Rešetnik, A. Terlević	28/06/2019	ZA-54960	0.326	0.001	5	4.70–5.09	2x	+
D158	Slano jezero	*D. sylvestris* subsp. *bertisceus*	Montenegro	900	42.75111	18.79916	S. Bogdanović, I. Ljubičić, I. Rešetnik, A. Terlević	29/06/2019	ZA-54964	0.334	0.001	5	3.55–3.60	2x	+
D169	Mt Shkelzeni	*D. sylvestris* subsp. *bertisceus*	Albania	2108	42.45568	20.11681	D. Shuka	24/07/2019	ZA-54975, ZA-54976	0.361	0.006	5	4.19–5.99	2x	+
D245	Bistra	*D. sylvestris* subsp. *bertisceus*	North Macedonia	1700	41.60719	20.75689	I. Rešetnik, M. Doboš, I. Ljubičić	20/07/2021	ZA-62635	0.344	0.007	4	4.20–5.84	2x	

D250	Grahovo road	*D. sylvestris* subsp. *bertisceus*	Montenegro	800	42.47019	18.85884	I. Rešetnik, M. Doboš, I. Ljubičić	24/07/2021	ZA-62639	0.347	0.008	4	4.52–5.63	2x	
D86	Sutjeska	*D. sylvestris* subsp. *bertisceus*	Bosnia and Herzegovina	638	43.31548	18.66821	S. Bogdanović, I. Ljubičić	11/07/2018	ZA-48662	0.330	0.003	4	2.55–5.47	2x	+
D87	Mt Maglic	*D. sylvestris* subsp. *bertisceus*	Bosnia and Herzegovina	1580	43.27309	18.71955	S. Bogdanović, I. Ljubičić	12/07/2018	ZA-48663	0.334	0.001	3	4.66–4.96	2x	+
D88	Piva river	*D. sylvestris* subsp. *bertisceus*	Montenegro	735	43.26326	18.84523	S. Bogdanović, I. Ljubičić	12/07/2018	ZA-48664	0.329	0.006	3	3.22–4.92	2x	+
D89	Pivska planina	*D. sylvestris* subsp. *bertisceus*	Montenegro	1680	43.13713	18.94089	S. Bogdanović, I. Ljubičić	13/07/2018	ZA-48665	0.331	0.006	3	2.15–4.03	2x	+
D90	Mt Durmitor	*D. sylvestris* subsp. *bertisceus*	Montenegro	2002	43.10045	19.05084	S. Bogdanović, I. Ljubičić	13/07/2018	ZA-48666	0.330	0.004	3	3.04–4.70	2x	+
D91	Berane	*D. sylvestris* subsp. *bertisceus*	Montenegro	711	42.88203	19.86147	S. Bogdanović, I. Ljubičić	13/07/2018	ZA-48612	0.328	0.002	3	3.03–4.01	2x	+
D92	Andrijevica	*D. sylvestris* subsp. *bertisceus*	Montenegro	746	42.72892	19.82458	S. Bogdanović, I. Ljubičić	13/07/2018	ZA-48667	0.325	0.003	3	3.04–3.69	2x	+
D93	Mt Prokletije Zeletin	*D. sylvestris* subsp. *bertisceus*	Montenegro	2011	42.64971	19.84412	S. Bogdanović, I. Ljubičić	14/07/2018	ZA-48668	0.330	0.007	3	2.71–3.78	2x	+
D94	Mt Prokletije Popadija W	*D. sylvestris* subsp. *bertisceus*	Montenegro	1989	42.52064	19.76142	S. Bogdanović, I. Ljubičić	14/07/2018	ZA-48669	0.333	0.009	3	2.96–3.97	2x	+
D95	Mt Prokletije Popadija SW	*D. sylvestris* subsp. *bertisceus*	Montenegro	1948	42.67997	20.01136	S. Bogdanović, I. Ljubičić	15/07/2018	ZA-48670	0.331	0.002	5	2.73–4.94	2x	+
D96	Moraca river	*D. sylvestris* subsp. *bertisceus*	Montenegro	278	42.69683	19.37431	S. Bogdanović, I. Ljubičić	15/07/2018	ZA-48613	0.332	0.007	3	4.01–4.60	2x	+
D178	Mt Kozjak	*D. sylvestris* subsp. *kozjakensis*	North Macedonia	1292	41.40914	21.67842	S. Bogdanović, I. Rešetnik	14/07/2019	ZA-54985	0.336	0.003	5	1.82–2.36	2x	+
D106	Sitnica	*D. sylvestris* subsp. *nodosus*	Bosnia and Herzegovina	915	42.56432	18.44312	S. Bogdanović, I. Ljubičić	17/7/2018, 29/6/2019	ZA-48623	0.328	0.002	5	1.79–5.00	2x	+
D124	Lastovo	*D. sylvestris* subsp. *nodosus*	Croatia	48	42.75519	16.92379	I. Rešetnik, S. Bogdanović	04/06/2019	ZA-54927	0.336	0.004	12	1.61–4.88	2x	
D127	Brac	*D. sylvestris* subsp. *nodosus*	Croatia	150	43.26608	16.69359	I. Ljubičić, A. Terlević, M. Grgurev	04/06/2019	ZA-54930	0.343	0.006	4	4.17–4.50	2x	
D128	Mosor	*D. sylvestris* subsp. *nodosus*	Croatia	810	43.52237	16.61158	S. Bogdanović, I. Ljubičić, I. Rešetnik, A. Terlević, M. Grgurev	05/06/2019	ZA-54931	0.337	0.004	5	2.73–4.41	2x	
D160	Peljesac	*D. sylvestris* subsp. *nodosus*	Croatia	160	42.91527	17.42674	S. Bogdanović, I. Ljubičić, I. Rešetnik, A. Terlević	29/06/2019	ZA-54966	0.331	0.005	5	4.28–5.03	2x	+
D183	Smricnjak	*D. sylvestris* subsp. *nodosus*	Croatia	83	43.75222	15.87861	L. Laca, S. Bogdanović	17/06/2020	ZA-56692	0.324	0.004	5	2.43–4.04	2x	+
D192	Vrsak	*D. sylvestris* subsp. *nodosus*	Croatia	1713	43.71589	16.8893	M. Doboš	22/07/2020	ZA-56821	0.331	0.002	5	2.61–4.10	2x	+
D193	Gornja Korita	*D. sylvestris* subsp. *nodosus*	Croatia	906	43.70998	16.8192	S. Bogdanović	22/07/2020	ZA-56822	0.337	0.009	5	3.93–5.57	2x	
D2	Korcula	*D. sylvestris* subsp. *nodosus*	Croatia	250	42.94086	17.08055	I. Rešetnik, S. Bogdanović	11/05/2018	ZA-46277	0.337	0.004	4	2.89–4.75	2x	
D254	Hrgud	*D. sylvestris* subsp. *nodosus*	Bosnia and Herzegovina	1069	43.09763	18.05759	I. Rešetnik, M. Doboš, I. Ljubičić	25/07/2021	ZA-62640	0.334	0.003	3	3.98–6.29	2x	
D27	Mt Velebit Plesevica	*D. sylvestris* subsp. *nodosus*	Croatia	1211	44.70794	14.96583	I. Rešetnik, S. Bogdanović, I. Ljubičić	15/06/2018	ZA-47427	0.335	0.007	3	2.88–4.93	2x	
D275	Hvar	*D. sylvestris* subsp. *nodosus*	Croatia	610	43.14478	16.5971	S. Bogdanović	28/07/2021	ZA-62642	0.348	0.003	5	3.20–5.61	2x	
D28	Mt Velebit Alan	*D. sylvestris* subsp. *nodosus*	Croatia	1020	44.7153	14.9569	I. Rešetnik, S. Bogdanović, I. Ljubičić	15/06/2018	ZA-47428	0.334	0.002	10	2.70–4.73	2x	+

D33	Mt Velebit Baske Ostarije	*D. sylvestris* subsp. *nodosus*	Croatia	905	44.52948	15.14316	I. Rešetnik, S. Bogdanović, I. Ljubičić	15/06/2018	ZA-47433	0.332	0.005	3	2.79–4.93	2x	+
D42	Mt Velebit Brundo	*D. sylvestris* subsp. *nodosus*	Croatia	1605	44.36112	15.51313	I. Rešetnik, S. Bogdanović, I. Ljubičić	17/06/2018	ZA-47442	0.344	0.004	5	3.00–6.03	2x	
D46	Sibenik	*D. sylvestris* subsp. *nodosus*	Croatia	18	43.73736	15.87176	I. Rešetnik, S. Bogdanović, I. Ljubičić	18/06/2018	ZA-47456	0.338	0.008	6	2.80–5.16	2x	+
D47-1	Mt Biokovo low	*D. sylvestris* subsp. *nodosus*	Croatia	325	43.25955	17.07843	I. Rešetnik, S. Bogdanović, I. Ljubičić	18/06/2018	ZA-47457	0.342	0.005	6	4.22–4.91	2x	+
D47-2	Mt Biokovo high	*D. sylvestris* subsp. *nodosus*	Croatia	1116	43.28407	17.08662	I. Rešetnik, S. Bogdanović, I. Ljubičić	18/06/2018	ZA-47459	0.344	0.009	7	2.75–5.73	2x	+
D52	Mostar	*D. sylvestris* subsp. *nodosus*	Bosnia and Herzegovina	382	43.32759	17.80412	I. Rešetnik, S. Bogdanović, I. Ljubičić	19/06/2018	ZA-47463	0.331	0.009	4	2.87–4.60	2x	+
D55	Konjic	*D. sylvestris* subsp. *nodosus*	Bosnia and Herzegovina	360	43.64998	17.96701	I. Rešetnik, S. Bogdanović, I. Ljubičić	19/06/2018	ZA-47466	0.332	0.001	6	2.24–4.75	2x	+
D56	Vranic	*D. sylvestris* subsp. *nodosus*	Bosnia and Herzegovina	549	43.41747	17.41643	I. Rešetnik, S. Bogdanović, I. Ljubičić	20/06/2018	ZA-47467	0.327	0.004	4	4.00–4.65	2x	+
D57	Blidinje	*D. sylvestris* subsp. *nodosus*	Bosnia and Herzegovina	1298	43.58668	17.48789	I. Rešetnik, S. Bogdanović, I. Ljubičić	20/06/2018	ZA-47468	0.334	0.004	5	2.49–5.07	2x	+
D6	Mironja	*D. sylvestris* subsp. *nodosus*	Croatia	332	42.81569	17.84349	I. Rešetnik, S. Bogdanović	13/5/2018, 29/6/2019	ZA-46281	0.325	0.003	9	2.60–4.85	2x	+
D60	Mt Cvrsnica Cavkarice	*D. sylvestris* subsp. *nodosus*	Bosnia and Herzegovina	1556	43.57896	17.54072	I. Rešetnik, S. Bogdanović, I. Ljubičić	20/06/2018	ZA-47471	0.346	0.008	4	2.72–4.92	2x	+
D61	Trobukva	*D. sylvestris* subsp. *nodosus*	Bosnia and Herzegovina	904	43.54695	17.27952	I. Rešetnik, S. Bogdanović, I. Ljubičić	20/06/2018	ZA-47472	0.331	0.001	4	1.36–3.96	2x	+
D63	Mt Cincar Krug	*D. sylvestris* subsp. *nodosus*	Bosnia and Herzegovina	1197	43.85963	17.03066	I. Rešetnik, S. Bogdanović, I. Ljubičić	21/06/2018	ZA-47474	0.334	0.006	5	1.84–2.76	2x	+
D64	Mt Cincar Begovaca	*D. sylvestris* subsp. *nodosus*	Bosnia and Herzegovina	1397	43.88544	17.05981	I. Rešetnik, S. Bogdanović, I. Ljubičić	21/06/2018	ZA-47475	0.336	0.006	4	3.95–4.70	2x	+
D65	Livno Jurina glava	*D. sylvestris* subsp. *nodosus*	Bosnia and Herzegovina	1096	43.78286	17.16035	I. Rešetnik, S. Bogdanović, I. Ljubičić	21/06/2018	ZA-47476	0.334	0.005	5	2.13–4.49	2x	+
D66	Drvar	*D. sylvestris* subsp. *nodosus*	Bosnia and Herzegovina	716	44.34866	16.35956	I. Rešetnik, S. Bogdanović, I. Ljubičić	22/06/2018	ZA-47477	0.340	0.002	3	4.52–4.99	2x	+
D8	Omis	*D. sylvestris* subsp. *nodosus*	Croatia	63	43.45025	16.69853	I. Rešetnik, S. Bogdanović	13/5/2018, 29/6/2019	ZA-46283	0.328	0.004	6	2.44–3.13	2x	+
D80	Mt Dinara Glavas	*D. sylvestris* subsp. *nodosus*	Croatia	740	44.01427	16.41704	S. Bogdanović, I. Ljubičić	30/06/2018	ZA-48607	0.340	0.008	6	4.28–6.00	2x	+
D82	Mt Svilaja	*D. sylvestris* subsp. *nodosus*	Croatia	934	43.75119	16.52322	S. Bogdanović, I. Ljubičić	01/07/2018	ZA-48608	0.336	0.003	5	3.53–4.66	2x	+
D85	Mt Vlasic	*D. sylvestris* subsp. *nodosus*	Bosnia and Herzegovina	1100	44.25852	17.62419	S. Bogdanović, I. Ljubičić	10/07/2018	ZA-48611	0.329	0.005	3	3.72–4.65	2x	+
D12	Nacinovici	*D. sylvestris* subsp. *sylvestris*	Croatia	240	45.12395	14.20468	S. Bogdanović, I. Ljubičić	19/5/2018, 13/7/2019	ZA-46416	0.641	0.007	8	2.43–4.66	4x*	+
D130	Krcic	*D. sylvestris* subsp. *sylvestris*	Croatia	345	44.03398	16.29247	S. Bogdanović, I. Ljubičić	14/06/2019	ZA-54933	0.327	0.002	5	2.48–3.89	2x	+
D162	Ostrc	*D. sylvestris* subsp. *sylvestris*	Croatia	740	45.76377	15.6486	A. Terlević, M. Grgurev	05/07/2019	ZA-54968	0.325	0.001	5	1.85–1.91	2x	+
D165	Litija	*D. sylvestris* subsp. *sylvestris*	Slovenia	295	46.08895	14.8873	A. Terlević, I. Ljubičić	09/07/2019	ZA-54971	0.331	0.003	5	3.31–4.29	2x	
D166	Polhograjska Grmada	*D. sylvestris* subsp. *sylvestris*	Slovenia	900	46.08407	14.33474	A. Terlević, I. Ljubičić	10/07/2019	ZA-54972	0.333	0.006	4	2.18–3.34	2x	+
D17	Mt Spacatto	*D. sylvestris* subsp. *sylvestris*	Italy	300	45.64227	13.8311	I. Rešetnik, S. Bogdanović, I. Ljubičić	13/06/2018	ZA-47417	0.640	0.004	3	2.00–2.54	4x	+
D184	Lim bay	*D. sylvestris* subsp. *sylvestris*	Croatia	136	45.13314	13.73433	A. Terlević	21/06/2020	ZA-56693	0.328	0.007	5	2.96–6.55	2x	+

D19-2	Vodice tetrapl	*D. sylvestris* subsp. *sylvestris*	Croatia	660	45.47917	14.04971	I. Rešetnik, S. Bogdanović, I. Ljubičić	13/6/2018, 13/7/2019	ZA-47419	0.651	0.006	8	1.80–3.57	4x	+
D196	Brkini	*D. sylvestris* subsp. *sylvestris*	Slovenia	625	45.50694	14.16806	B. Frajman	15/08/2020	ZA-56825	0.657	0.006	5	1.85–2.80	4x	
D20	Zbevnica	*D. sylvestris* subsp. *sylvestris*	Croatia	941	45.45721	14.01632	I. Rešetnik, S. Bogdanović, I. Ljubičić	13/6/2018, 19/7/2020	ZA-47420	0.651	0.004	3	3.55–4.50	4x*	+
D205	Klecice	*D. sylvestris* subsp. *sylvestris*	Croatia	968	45.26685	15.1413	I. Ljubičić, A. Terlević	16/06/2021	ZA-62605	0.337	0.007	4	4.36–5.37	2x	
D206	Mt Calvo	*D. sylvestris* subsp. *sylvestris*	Italy	360	45.65316	13.82246	I. Rešetnik, S. Bogdanović, M. Temunović, A. Terlević	28/06/2021	ZA-62606	0.646	0.008	4	2.57–3.58	4x	
D207	Hervati	*D. sylvestris* subsp. *sylvestris*	Italy	345	45.6259	13.86941	I. Rešetnik, S. Bogdanović, M. Temunović, A. Terlević	28/06/2021	ZA-62607	0.652	0.009	6	2.70–4.30	4x	
D21	Slum	*D. sylvestris* subsp. *sylvestris*	Croatia	553	45.4234	14.01213	I. Rešetnik, S. Bogdanović, I. Ljubičić	13/6/2018, 19/7/2020	ZA-47421	0.650	0.009	4	2.26–5.01	4x*	+
D22	Mt Ucka	*D. sylvestris* subsp. *sylvestris*	Croatia	525	45.31681	14.17734	I. Rešetnik, S. Bogdanović, I. Ljubičić	13/06/2018	ZA-47422	0.656	0.010	4	2.02–4.90	4x	+
D24	Gornje Jelenje	*D. sylvestris* subsp. *sylvestris*	Croatia	862	45.36157	14.61724	I. Rešetnik, S. Bogdanović, I. Ljubičić	14/06/2018	ZA-47424	0.648	0.003	5	2.35–4.87	4x	+
D256	Velez	*D. sylvestris* subsp. *sylvestris*	Bosnia and Herzegovina	1912	43.35755	17.93495	I. Rešetnik, M. Doboš, I. Ljubičić	25/07/2021	ZA-62641	0.340	0.009	4	4.23–5.21	2x	
D34	Mt Velebit Visocica	*D. sylvestris* subsp. *sylvestris*	Croatia	1458	44.4293	15.3643	I. Rešetnik, S. Bogdanović, I. Ljubičić	16/06/2018	ZA-47434	0.334	0.004	4	3.14–4.43	2x	
D37	Mt Velebit Buljma	*D. sylvestris* subsp. *sylvestris*	Croatia	1218	44.36597	15.46143	I. Rešetnik, S. Bogdanović, I. Ljubičić	17/06/2018	ZA-47437	0.336	0.004	5	3.77–4.93	2x	+
D38	Mt Paklenica Struge	*D. sylvestris* subsp. *sylvestris*	Croatia	1368	44.37109	15.46567	I. Rešetnik, S. Bogdanović, I. Ljubičić	17/06/2018	ZA-47438	0.339	0.006	3	2.74–4.53	2x	+
D58	Mt Cvrsnica SW	*D. sylvestris* subsp. *sylvestris*	Bosnia and Herzegovina	1928	43.59503	17.54076	I. Rešetnik, S. Bogdanović, I. Ljubičić	20/06/2018	ZA-47469	0.325	0.001	3	2.97–4.59	2x	
D59	Mt Cvrsnica Siroka kosa	*D. sylvestris* subsp. *sylvestris*	Bosnia and Herzegovina	1732	43.58652	17.53378	I. Rešetnik, S. Bogdanović, I. Ljubičić	20/06/2018	ZA-47470	0.334	0.007	4	3.72–5.07	2x	+
D81	Mt Dinara Osljak	*D. sylvestris* subsp. *sylvestris*	Croatia	1418	44.03867	16.39343	S. Bogdanović, I. Ljubičić	30/06/2018	ZA-48606	0.336	0.008	3	4.39–4.93	2x	+
D83	Banja Luka	*D. sylvestris* subsp. *sylvestris*	Bosnia and Herzegovina	207	44.68547	17.1797	S. Bogdanović, I. Ljubičić	10/07/2018	ZA-48609	0.329	0.005	3	2.99–4.99	2x	+
D84	Jajce	*D. sylvestris* subsp. *sylvestris*	Bosnia and Herzegovina	380	44.3468	17.24253	S. Bogdanović, I. Ljubičić	10/07/2018	ZA-48610	0.338	0.009	3	2.12–4.87	2x	
D1	Klek village	*D. sylvestris* subsp. *tergestinus*	Croatia	80	42.94874	17.56451	I. Rešetnik, S. Bogdanović	09/05/2018	ZA-46276	0.345	0.005	3	4.58–5.00	2x	+
D11	Gornji Kamenjak	*D. sylvestris* subsp. *tergestinus*	Croatia	55	44.81097	13.90088	S. Bogdanović, I. Ljubičić	17/05/2018	ZA-46415	0.339	0.005	4	2.96–4.68	2x	+
D111	Cres	*D. sylvestris* subsp. *tergestinus*	Croatia	40	44.97721	14.445	S. Bogdanović, S. Cambria	21/10/2018	ZA-48628	0.342	0.002	4	2.05–4.25	2x	
D129	Gradina	*D. sylvestris* subsp. *tergestinus*	Croatia	186	43.77399	15.95608	S. Bogdanović, I. Ljubičić, I. Rešetnik, A. Terlević, M. Grgurev	05/06/2019	ZA-54932	0.340	0.004	4	2.98–4.02	2x	+
D13	Krizisce	*D. sylvestris* subsp. *tergestinus*	Croatia	513	45.2676	14.59441	S. Bogdanović, I. Ljubičić	19/05/2018	ZA-46417	0.333	0.004	4	3.75–4.68	2x	+
D16	Sezana	*D. sylvestris* subsp. *tergestinus*	Italy	344	45.70153	13.83729	I. Rešetnik, S. Bogdanović, I. Ljubičić	13/06/2018	ZA-47416	0.341	0.007	4	3.31–3.82	2x	+
D18	Petrinje	*D. sylvestris* subsp. *tergestinus*	Slovenia	422	45.57284	13.90681	I. Rešetnik, S. Bogdanović, I. Ljubičić	13/06/2018	ZA-47418	0.336	0.008	5	2.66–4.99	2x	+
D185	Krk	*D. sylvestris* subsp. *tergestinus*	Croatia	220	45.02389	14.64694	I. Rešetnik	14/06/2020	ZA-56694	0.340	0.004	5	3.30–6.93	2x*	

D25	Bunica	*D. sylvestris* subsp. *tergestinus*	Croatia	8	45.02484	14.88473	I. Rešetnik, S. Bogdanović, I. Ljubičić	14/06/2018	ZA-47425	0.337	0.001	4	2.67–4.62	2x	
D29	Donji Bileni	*D. sylvestris* subsp. *tergestinus*	Croatia	274	44.71196	14.9225	I. Rešetnik, S. Bogdanović, I. Ljubičić	15/06/2018	ZA-47429	0.340	0.007	5	2.46–4.92	2x	
D32	Karlobag	*D. sylvestris* subsp. *tergestinus*	Croatia	12	44.53338	15.06519	I. Rešetnik, S. Bogdanović, I. Ljubičić	15/06/2018	ZA-47432	0.335	0.005	3	4.09–4.66	2x*	
D36	Mt Velebit Paklenica	*D. sylvestris* subsp. *tergestinus*	Croatia	397	44.31347	15.45328	I. Rešetnik, S. Bogdanović, I. Ljubičić	17/06/2018	ZA-47436	0.340	0.006	4	2.86–4.77	2x	+
D4	Dubrovnik	*D. sylvestris* subsp. *tergestinus*	Croatia	279	42.64521	18.12021	I. Rešetnik, S. Bogdanović	12/05/2018	ZA-46279	0.352	0.005	3	2.75–4.26	2x	+
D43	Vransko jezero	*D. sylvestris* subsp. *tergestinus*	Croatia	35	43.86523	15.6423	I. Rešetnik, S. Bogdanović, I. Ljubičić	18/06/2018	ZA-47453	0.341	0.008	4	2.58–4.72	2x	
D50	Mostar Vukodol	*D. sylvestris* subsp. *tergestinus*	Bosnia and Herzegovina	250	43.32542	17.79292	I. Rešetnik, S. Bogdanović, I. Ljubičić	19/06/2018	ZA-47461	0.353	0.005	5	3.32–4.18	2x	
D53	Mostar Hum	*D. sylvestris* subsp. *tergestinus*	Bosnia and Herzegovina	385	43.32755	17.80407	I. Rešetnik, S. Bogdanović, I. Ljubičić	19/06/2018	ZA-47464	0.355	0.009	5	2.11–4.87	2x	
D62	Livno	*D. sylvestris* subsp. *tergestinus*	Bosnia and Herzegovina	821	43.84762	16.9733	I. Rešetnik, S. Bogdanović, I. Ljubičić	21/06/2018	ZA-47473	0.340	0.008	3	2.81–4.33	2x	+
D7	Mravinica Doli	*D. sylvestris* subsp. *tergestinus*	Croatia	351	42.81084	17.83079	I. Rešetnik, S. Bogdanović	13/05/2018	ZA-46282	0.351	0.007	3	2.49–4.33	2x	+
D9	Malacka	*D. sylvestris* subsp. *tergestinus*	Croatia	602	43.58147	16.32579	I. Rešetnik, S. Bogdanović	13/05/2018	ZA-46284	0.339	0.003	3	4.57–5.88	2x	+
16866	Ahrntal	*D. sylvestris* s.l. - Alps	Italy	1075	46.89278	11.96194	D. Volgger, F. Faltner, B. Frajman	12/07/2021	IB-16866	0.366	0.001	3	5.99–8.21	2x	
16867	Pustertal Niederdorf	*D. sylvestris* s.l. - Alps	Italy	1850	46.70778	12.17528	D. Volgger, A. Seyer, B. Frajman	12/07/2021	IB-16867	0.352	0.010	3	2.71–3.08	2x	
16868	Karnische Alpen	*D. sylvestris* s.l. - Alps	Austria	1960	46.69444	12.49306	D. Volgger, B. Frajman	13/07/2021	IB-16868	0.326	0.005	3	4.30–5.60	2x	
16869	Dolomiti Passo Tre Croci	*D. sylvestris* s.l. - Alps	Italy	1810	46.5575	12.19944	D. Volgger, B. Frajman	13/07/2021	IB-16869	0.330	0.004	3	4.89–9.03	2x	
16870	Dolomiti Fiames	*D. sylvestris* s.l. - Alps	Italy	1410	46.59667	12.11222	D. Volgger, B. Frajman	13/07/2021	IB-16870	0.334	0.001	3	4.80–8.79	2x	
16871	Dolomiti Furkelpass	*D. sylvestris* s.l. - Alps	Italy	2140	46.71139	11.97028	B. Frajman	13/07/2021	IB-16871	0.354	0.003	3	6.76–7.30	2x	
16872	Pustertal Issing	*D. sylvestris* s.l. - Alps	Italy	870	46.81	11.84306	D. Volgger, B. Frajman	13/07/2021	IB-16872	0.359	0.004	3	6.80–9.45	2x	
16875	Pustertal Burgkofl	*D. sylvestris* s.l. - Alps	Italy	935	46.79278	11.88111	F. Faltner	12/07/2021	IB-16875	0.348	0.008	3	8.83–10.19	2x	
16927	Villgrater Berge	*D. sylvestris* s.l. - Alps	Austria	1500	46.80917	12.33083	B. Frajman, M. Doboš	02/08/2021	IB-16927	0.339	0.001	5	2.34–5.62	2x	
16972	Pordenone	*D. sylvestris* s.l. - Alps	Italy	900	46.28	12.36778	P. Schönswetter, T. Zeni	31/07/2021	IB-16972	0.346	0.006	5	3.81–5.44	2x	
16973	Prealpi Bellunesi	*D. sylvestris* s.l. - Alps	Italy	1515	46.11278	12.50778	P. Schönswetter, T. Zeni	01/08/2021	IB-16973	0.328	0.001	3	6.65–9.27	2x	
16977	Dolomiti Strudelkopf	*D. sylvestris* s.l. - Alps	Italy	2170	46.64417	12.2	A. Seyr	13/08/2021	IB-16977	0.335	0.005	6	3.82–4.90	2x	
D121	Oberes Gericht	*D. sylvestris* s.l. - Alps	Austria	1160	47.03669	10.64664	M. Falch	16/07/2018	ZA-54924	0.351	0.003	3	2.47–3.43	2x	
D122	Innsbruck S	*D. sylvestris* s.l. - Alps	Austria	880	47.24425	11.41853	M. Falch	03/09/2018	ZA-54925	0.360	0.004	3	1.87–4.49	2x	
D164	Vrsic	*D. sylvestris* s.l. - Alps	Slovenia	1954	46.43132	13.73659	A. Terlević, I. Ljubičić	09/07/2019	ZA-54970	0.333	0.000	6	2.24–2.87	2x	
D197	Achberg	*D. sylvestris* s.l. - Alps	Austria	785	47.28972	10.98667	B. Frajman	07/07/2020	ZA-56826	0.359	0.004	5	2.49–6.76	2x	

D199	Gailtal Arnoldstein	*D. sylvestris* s.l. - Alps	Austria	545	46.56528	13.69083	B. Frajman, P. Schönswetter	22/05/2020	ZA-56828	0.333	0.002	5	2.62–6.11	2x	
D67	Prutz	*D. sylvestris* s.l. - Alps	Austria	904	47.07507	10.65361	I. Rešetnik	27/06/2018	ZA-47478	0.363	0.008	3	1.94–4.48	2x	
D68	Mals	*D. sylvestris* s.l. - Alps	Italy	1039	46.67989	10.55889	I. Rešetnik	27/06/2018	ZA-47479	0.361	0.002	3	2.04–3.96	2x	
D69	Gailtal alps	*D. sylvestris* s.l. - Alps	Austria	1971	46.75922	12.88028	I. Rešetnik	29/06/2018	ZA-47480	0.335	0.005	5	1.93–2.89	2x	
D70	Zentralalpen	*D. sylvestris* s.l. - Alps	Austria	2030	47.01306	12.25111	I. Rešetnik	30/06/2018	ZA-47481	0.333	0.004	3	2.48–2.90	2x	
D71	Innsbruck Nordkette	*D. sylvestris* s.l. - Alps	Austria	1906	47.30494	11.35861	I. Rešetnik	08/07/2018	ZA-47482	0.358	0.005	3	2.51–4.57	2x	
Lago di Fedaia	Lago di Fedaia	*D. sylvestris* s.l. - Alps	Italy	2220	46.46606	11.85929	H. Luqman	04/08/2016	ZT-Lago di Fedaia	0.359	0.000	3	6.69–9.79	2x	
Mt Caslano	Mt Caslano	*D. sylvestris* s.l. - Alps	Switzerland	332	45.96053	8.88331	H. Luqman	26/06/2017	ZT-Mt Caslano	0.355	0.005	3	6.86–7.55	2x	
Mustair Low	Mustair Low	*D. sylvestris* s.l. - Alps	Switzerland	1300	46.63216	10.44699	H. Luqman	03/08/2017	ZT-Mustair Low	0.366	0.003	3	4.89–5.81	2x	
Rifugio Graziani	Rifugio Graziani	*D. sylvestris* s.l. - Alps	Italy	1566	45.79879	10.89384	H. Luqman	15/07/2017	ZT-Rifugio Graziani	0.353	0.005	3	3.81–4.64	2x	
Schwarzenmatt	Schwarzenmatt	*D. sylvestris* s.l. - Alps	Switzerland	1220	46.63409	7.349496	H. Luqman	23/07/2016	ZT-Schwarzenmatt	0.369	0.004	3	5.28–7.11	2x	
Thumel	Thumel	*D. sylvestris* s.l. - Alps	Italy	1940	45.53495	7.10119	H. Luqman	26/08/2017	ZT-Thumel	0.367	0.008	3	3.98–4.75	2x	
D102	Obzovica	*D. sylvestris* s.l. - Balkan	Montenegro	931	42.30581	18.93189	S. Bogdanović, I. Ljubičić	16/07/2018	ZA-48619	0.335	0.001	4	2.82–4.14	2x	+
D103	Mt Lovcen	*D. sylvestris* s.l. - Balkan	Montenegro	1681	42.4003	18.83807	S. Bogdanović, I. Ljubičić	16/07/2018	ZA-48620	0.337	0.007	4	3.95–4.88	2x	+
D108	Gramsh	*D. sylvestris* s.l. - Balkan	Albania	969	40.86694	20.38444	B. Frajman, P. Schönswetter	12/07/2018	ZA-48625	0.336	0.002	4	4.19–5.26	2x	
D109	Maja e Ostrovice	*D. sylvestris* s.l. - Balkan	Albania	1406	40.57972	20.47722	B. Frajman, P. Schönswetter	13/07/2018	ZA-48626	0.336	0.007	3	2.92–4.93	2x	
D110	Mt Jablanica	*D. sylvestris* s.l. - Balkan	North Macedonia	1759	41.27278	20.535	B. Frajman, P. Schönswetter	15/07/2018	ZA-48627	0.342	0.003	3	2.94–4.92	2x	
D119	Mt Valamara	*D. sylvestris* s.l. - Balkan	Albania	1670	40.82167	20.50167	M. Duchon	16/07/2018	ZA-54922	0.351	0.008	3	1.70–4.23	2x	
D145	Mat	*D. sylvestris* s.l. - Balkan	Albania	145	41.67954	19.84739	S. Bogdanović, I. Ljubičić, I. Rešetnik, A. Terlević	26/06/2019	ZA-54947	0.338	0.007	5	3.73–4.76	2x	+
D147	Shkoder	*D. sylvestris* s.l. - Balkan	Albania	129	42.07219	19.56423	S. Bogdanović, I. Ljubičić, I. Rešetnik, A. Terlević	27/06/2019	ZA-54953	0.330	0.002	8	3.16–4.50	2x	+
D170	Mt Kopaonik	*D. sylvestris* s.l. - Balkan	Serbia	1787	43.26151	20.83653	S. Bogdanović, M. Doboš, M. Grgurev, I. Rešetnik	10/07/2019	ZA-54977	0.334	0.001	6	2.14–2.48	2x	+
D171	Ljuboten peak	*D. sylvestris* s.l. - Balkan	North Macedonia	2255	42.19948	21.12385	S. Bogdanović, M. Doboš, M. Grgurev, I. Rešetnik	11/07/2019	ZA-54978	0.335	0.005	5	1.83–4.03	2x	+
D172	Ljuboten peak scardicus	*D. sylvestris* s.l. - Balkan	North Macedonia	2255	42.19948	21.12385	S. Bogdanović, M. Doboš, M. Grgurev, I. Rešetnik	11/07/2019	ZA-54979	0.336	0.004	5	2.31–4.65	2x	+
D173	Mt Bistra	*D. sylvestris* s.l. - Balkan	North Macedonia	1687	41.6293	20.68481	S. Bogdanović, M. Doboš, M. Grgurev, I. Rešetnik	12/07/2019	ZA-54980	0.336	0.001	6	1.86–2.17	2x	+
D174	Mt Galicica	*D. sylvestris* s.l. - Balkan	North Macedonia	1510	40.96566	20.81879	S. Bogdanović, M. Doboš, M. Grgurev, I. Rešetnik	12/07/2019	ZA-54981	0.339	0.000	5	2.37–4.61	2x	+
D175	Mt Korab Nistrovski	*D. sylvestris* s.l. - Balkan	North Macedonia	2112	41.7919	20.59291	S. Bogdanović, M. Doboš, M. Grgurev, I. Rešetnik	13/07/2019	ZA-54982	0.337	0.004	5	2.98–4.02	2x	+
D176	Mt Korab Radika	*D. sylvestris* s.l. - Balkan	North Macedonia	1379	41.79959	20.62801	S. Bogdanović, M. Doboš, M. Grgurev, I. Rešetnik	13/07/2019	ZA-54983	0.337	0.001	5	2.39–2.41	2x	+

D179	Mt Jakupica Solunska glava	*D. sylvestris* s.l. - Balkan	North Macedonia	2274	41.69279	21.40381	S. Bogdanović, M. Grgurev, I. Rešetnik	17/07/2019	ZA-54986	0.347	0.005	5	2.02–3.73	2x	+
D180	Mt Jakupica Ismailica	*D. sylvestris* s.l. - Balkan	North Macedonia	1235	41.68044	21.43242	S. Bogdanović, M. Grgurev, I. Rešetnik	17/07/2019	ZA-54987	0.345	0.006	5	1.75–2.61	2x	+
D182	Gjergjevica	*D. sylvestris* s.l. - Balkan	Albania	1300	40.58626	20.57514	L. Shuka	27/07/2019	ZA-54989, ZA-54990	0.376	0.004	4	4.73–6.00	2x	+
D204	Voskopoje	*D. sylvestris* s.l. - Balkan	Albania	1165	40.58247	20.56072	D. Lakušić, N. Kuzmanović, I. Janković, M. Zbiljić	11/07/2018	ZA-58876	0.355	0.004	3	5.63–8.89	2x	
D246	Sar Planina	*D. sylvestris* s.l. - Balkan	Albania	2119	42.09306	20.8931	I. Rešetnik, M. Doboš, I. Ljubičić	21/07/2021	ZA-62636	0.338	0.006	3	3.26–4.52	2x	
D248	Mali Gropa	*D. sylvestris* s.l. - Balkan	Albania	1616	41.37702	20.06664	I. Rešetnik, M. Doboš, I. Ljubičić	22/07/2021	ZA-62637	0.348	0.003	4	3.85–5.21	2x	
D76	Maljen	*D. sylvestris* s.l. - Balkan	Serbia	640	44.04424	20.01645	S. Đurović, G. Tomović	15/07/2018	ZA-48631	0.328	0.006	4	2.95–4.37	2x	
D77	Mt Sar Planina	*D. sylvestris* s.l. - Balkan	Serbia	1755	42.2335	20.91537	D. Lakušić, N. Kuzmanović, I. Janković, M. Zbiljić	14/07/2018	ZA-48632	0.340	0.004	3	2.23–4.34	2x	
D79	Mt Kopaonik Pancicev vrh	*D. sylvestris* s.l. - Balkan	Serbia	1660	43.26149	20.83973	S. Đurović, G. Tomović	13/07/2018	ZA-48634	0.335	0.004	3	3.69–3.95	2x	

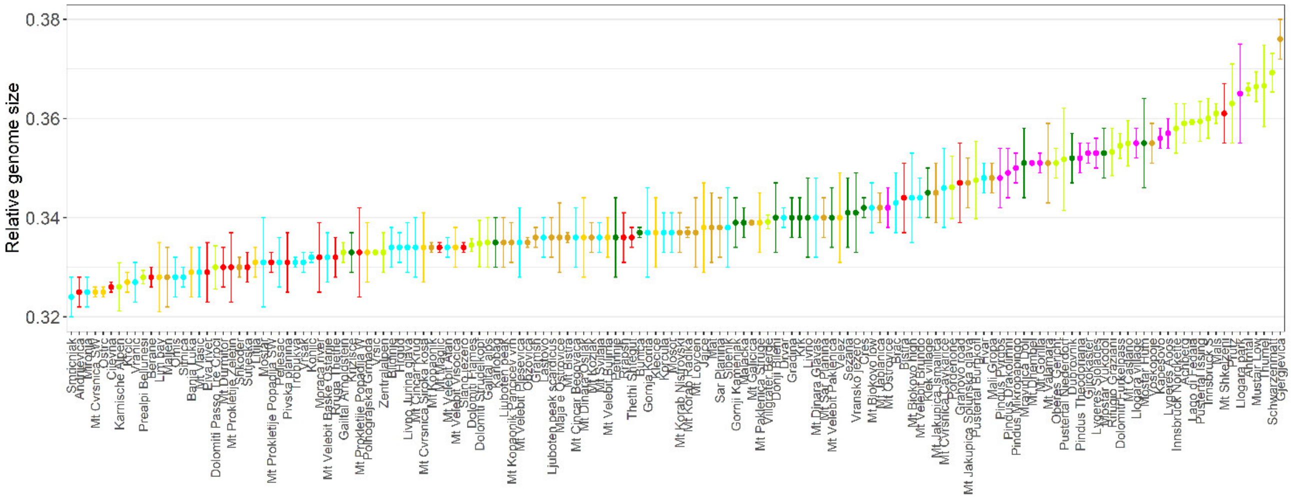

Figure S1. Relative genome size (RGS) in diploid populations of *Dianthus sylvestris* sorted by increasing RGS values. Population means (dots) with corresponding standard deviation (vertical lines) are presented. Population names correspond to Table S1. Colors correspond to subspecies, as in Figure 2.

Table S2. Results of Kruskal Wallis test between diploid (2*x*, N = 9) and tetraploid (4*x*, N = 7) populations of *Dianthus sylvestris* subsp. *sylvestris*. Morphological characters with significant differences between ploidy levels at $p < 0.05$ are shown in bold.

**Character**	**Abbreviation**	**statistic**	***p* value**
Length of the woody part of the stem below basal leaves	BL	0.025779	0.872442
Basal leaf length	BLL	4.495013	**0.033994**
Calyx length	CL	0.02521	0.873845
Length of the cauline leaf from the second stem node	CLL	6.463286	**0.011013**
Calyx teeth length	CTL	1.235294	0.26638
Number of epicalyx scales	ESN	1.153723	0.282771
Height of first branching	FBH	7.868347	**0.005031**
Number of flowers per stem	FPS	2.072495	0.149976
Inflorescence length	IL	2.876809	0.089864
Inner epicalyx scale apex length	ISAL	0.809524	0.368261
Inner epicalyx scale length	ISL	1.235294	0.26638
Inner epicalyx scale width	ISW	2.691877	0.100861
Length of the first stem internode	LIL	0.002818	0.957667
Plant height	PH	5.9359	**0.014835**
Petal length	PL	0.226891	0.633839
Petal limb length	PLL	0.02521	0.873845
Petal limb width	PLW	0.02521	0.873845
Petal teeth length	PTL	0.630252	0.427263
Maximum number of petal teeth	PTMAX	0.912933	0.339337
Minimum number of petal teeth	PTMIN	2.707805	0.099858
Ratio Calyx teeth length / Calyx length	RCALYX	2.699817	0.10036
Ratio Petal teeth length / Petal limb length	RDENT	0.280938	0.596087

Ratio Calyx length / Claw length	RFL	1.011204	0.314614
Ratio Inner epicalyx scale length without apex / Inner epicalyx scale width	RIS	1.481793	0.223494
Ratio Inner epicalyx scale apex length / Inner epicalyx scale length	RISA	0.070028	0.791296
Ratio Petal limb length / Petal limb width	RLIMB	1.122098	0.289467
Ratio Petal limb length / Petal length	RPETAL	0.718143	0.396754
Length of the last stem internode below branching	UIL	4.715618	**0.02989**

Table S3. Results of Kruskal Wallis test between diploid (2*x*, N = 15) and tetraploid (4*x*, N = 10) populations of *Dianthus sylvestris* subsp. *sylvestris*. Environmental variables with significant differences between ploidy levels at $p < 0.05$ are shown in bold.

short name	long name	unit	statistic	*p* value
bio01	mean annual air temperature	°C/10	5.060548	**0.024477**
bio02	mean diurnal air temperature range	°C/10	4.601475	**0.031944**
bio03	isothermality	°C/10	5.829034	**0.015764**
bio04	temperature seasonality	°C/10	0.249231	0.617617
bio05	mean daily maximum air temperature of the warmest month	°C/10	0.943033	0.331499
bio06	mean daily minimum air temperature of the coldest month	°C/10	9.484988	**0.002072**
bio07	annual range of air temperature	°C/10	3.673611	0.05528
bio08	mean daily mean air temperatures of the wettest quarter	°C/10	1.492675	0.221802
bio09	mean daily mean air temperatures of the driest quarter	°C/10	0.249327	0.61755
bio10	mean daily mean air temperatures of the warmest quarter	°C/10	4.681801	**0.030484**
bio11	mean daily mean air temperatures of the coldest quarter	°C/10	6.533385	**0.010587**
bio12	annual precipitation amount	kg m^{-2}	0.062332	0.802848
bio13	precipitation amount of the wettest month	kg m^{-2}	0.197151	0.657031
bio14	precipitation amount of the driest month	kg m^{-2}	5.198299	**0.022609**
bio15	precipitation seasonality	kg m^{-2}	2.972931	0.084668
bio16	mean monthly precipitation amount of the wettest quarter	kg m^{-2}	0.249231	0.617617
bio17	mean monthly precipitation amount of the driest quarter	kg m^{-2}	4.102386	**0.042823**
bio18	mean monthly precipitation amount of the warmest quarter	kg m^{-2}	0.603309	0.437318
bio19	mean monthly precipitation amount of the coldest quarter	kg m^{-2}	0.012308	0.911664
dtm_east	eastness	index	8.972308	**0.002741**
dtm_north	northness	index	0.110769	0.73927
dtm_slope	slope	°	4.68	**0.030516**
nfd	number of frost days	No days	9.833654	**0.001713**
sdays	number of snow days	No days	7.240477	**0.007128**
soil_clay	clay content	%	6.484245	**0.010883**
soil_org_carb	soil organic carbon content	x 5 g kg^{-1}	1.563563	0.211144
soil_ph_h2o	soil pH x 10 in H2O	pH	1.238427	0.265774
soil_sand	sand content	%	1.236239	0.266197
soil_water	soil water content at 33kPa		0.625926	0.428854
srad	surface solar radiation downwards	kJ m^{-2}	4.68	**0.030516**

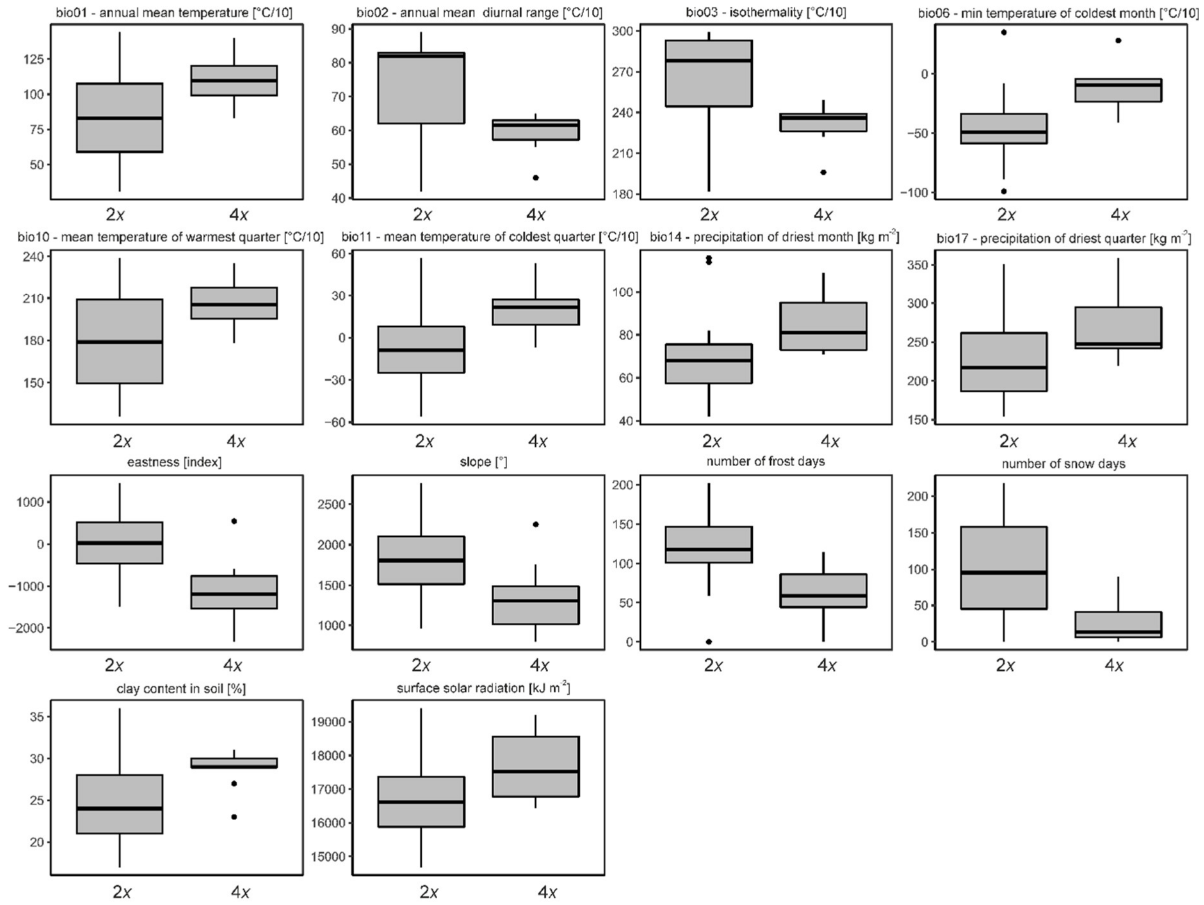

Figure S2. Boxplots showing environmental differences along the 14 environmental variables between diploid (2*x*) and tetraploid (4*x*) populations of *Dianthus sylvestris* subsp. *sylvestris*. Only variables with significant differences ($p < 0.05$), as revealed by Kruskal Wallis test (Table S3), are shown.
